# DNA Methylation in Osteoarthritis: Current Status and Therapeutic Implications

**DOI:** 10.2174/1874312901812010037

**Published:** 2018-03-30

**Authors:** Antonio Miranda-Duarte

**Affiliations:** Department of Genetics, Instituto Nacional de Rehabilitación “Luis Guillermo Ibarra Ibarra”, Tlalpan, Mexico

**Keywords:** Osteoarthritis, Epigenetics, DNA, Methylation, Cartilage, Chondrocyte

## Abstract

**Background:**

Primary Osteoarthritis (OA) is a multifactorial disease in which genetic factors are strongly associated with its development; however, recently it has been observed that epigenetic modifications are also involved in the pathogenesis of OA. DNA methylation is related to gene silencing, and several studies have investigated its role in the *loci* of different pathways or molecules associated to OA.

**Objective:**

This review is focused on the current status of DNA methylation studies related to OA pathogenesis.

**Method:**

A review of the literature was conducted on searching in PUBMED for original papers on DNA methylation in OA.

**Conclusion:**

The DNA methylation research of *loci* related to OA pathogenesis has shown a correlation between methylation and gene repression; however, there are some exceptions to this rule. Recently, the development of genome-wide methylation and genome-wide hydroxymethylation profiles has demonstrated that several genes previously associated with OA can have changes in their methylation status, favoring the development of the disease, and these have even shown the role of other epigenetic markers.

## 1 INTRODUCTION

Osteoarthritis (OA) continues to be considered the most common joint disease and a leading cause of musculoskeletal disability worldwide. Its hallmark is the progressive degeneration of articular cartilage, resulting in joint space narrowing, osteophyte formation, and subchondral sclerosis that, taken together, are clinically translated as chronic joint pain, joint stiffness, limitation of movement, and a variable degree of inflammation [1]. OA is a complex disease in which genetics and environmental factors are strongly related to its development. Generally, it is classified as primary or idiopathic when no discernible cause is evident, and secondary when a triggering factor is apparent. Idiopathic OA, in particular, possesses a significant genetic component, and several genetic association studies have demonstrated its relationship with different genes, such as those involved with inflammation, Wnt signaling, Bone Morphogenetic Proteins (BMP), proteases, and Extracellular Matrix (ECM) proteins, among others [2]. However, there has not always been consistency in the results, probably due to different gene–gene and gene–environment interactions. 

Epigenetics refers to heritable changes in gene expression that occur without changes in the DNA, and it comprises a mechanism through which gene–environment interactions could occur. Its mechanisms include DNA methylation, histone modifications, chromatin remodeling, and microRNAs (miRNA) [3]. Recent evidence has shown that epigenetic changes could affect the expression of genes involved in the pathogenesis of OA; this review is focused on the current state of DNA methylation studies related to OA pathogenesis.

## 2 METHODS

A PUBMED literature review was conducted searching for original papers on DNA methylation in OA, using the key words “osteoarthritis”, “DNA methylation”, “articular cartilage”, and “chondrocyte”.

## 3 RESULT

### 3.1 Articular Cartilage and the Pathogenesis of Osteoarthritis

A review of the pathogenesis of OA falls beyond the scope of this paper; however, the most important aspects are mentioned briefly. Articular cartilage is a highly specialized, avascular, and aneural connective tissue composed of ECM and chondrocytes. ECM is composed mainly of collagens and proteoglycans, among which type II collagen is the major constituent of ECM; this forms fibrils and fibers intertwined with proteoglycan aggregates. Other collagens, such as types I, IV, V, VI, IX, and XI, are present in a minor proportion. The second major component comprises the proteoglycan aggrecan; this interacts with hyaluronan to form aggregates and occupies the ECM interfibrillar space [4]. Chondrocytes are the unique cellular element of articular cartilage and, under physiological conditions, are responsible for a subtle balance between ECM synthesis and degradation. These cells are produced during chondrogenesis, which is the cartilage differentiation process that leads endochondral ossification to skeletal formation. The chondrogenesis process consists of the condensation of Mesenchymal Stem Cells (MSC), differentiation into chondrocytes, proliferation, and hypertrophic maturation. Proliferating chondrocytes are characterized by the expression of collagens type II, IX, and XII, and aggrecan [4], and when they become hypertrophic, they are characterized by the production of type X collagen. Eventually, terminally differentiated chondrocytes undergo apoptosis and the ECM is mineralized and replaced by bone [5].

In OA, the balance between synthesis and degradation conferred by chondrocytes is lost, resulting in cartilage destruction. Once the osteoarthritic process has begun, phenomena such as proliferation and hypertrophic differentiation of chondrocytes, remodeling, ECM mineralization, and apoptosis also occur [6]. At the onset of OA, the quantity and composition of ECM undergo a change. There is a significantly reduced synthesis of aggrecan and an increase of collagen synthesis, but with a modification in collagen-type composition, changing from collagen type II to collagen type I, which is more characteristic of subchondral bone. To date, it is well-recognized that OA possesses an important inflammatory component. Inflammation is a triggering factor for OA and leads chondrocytes and synovium to produce cytokines such as Interleukin-1Beta (IL-1β) and Tumor Necrosis Factor α (TNF-α), among others [7, 8]. These inflammatory cytokines contribute to a catabolic process and favor the synthesis of Matrix Metalloproteinases (MMP) and A Disintegrin And Metalloproteinase Thrombospondin type I Motifs (ADAMTS), with MMP13 and ADAMTS5 the principal collagenase and aggrecanase, respectively [9, 10]. On the other hand, anabolic cytokines Insulin-like Growth Factor 1 (IGF-1), Transforming Growth Factor β1 (TGF-β1), 2, and 3, Fibroblast Growth Factors (FGF) 2, 4, and 8, and BMP stimulate ECM synthesis in an attempt to repair the damage [7, 8]. However, the degenerative activity of matrix-degrading proteins is enhanced by the increased levels of Nitric Oxide (NO), which is also upregulated by inflammatory cytokines. In turn, NO upregulates the transcriptional activity of the Nuclear Factor kappa-light-chain-enhancer of activated Beta cells (NF-κB), maintaining chronic inflammation, mediating apoptosis of chondrocytes and perpetuating articular cartilage damage [7].

### 3.2 DNA Methylation

The most widely studied epigenetic modification in humans is DNA methylation that, in general, is correlated with gene silencing. DNA methylation occurs in CpG dinucleotides, which are quite rare in the human genome because their frequency is approximately 1%. These dinucleotides are clustered in islands, which are regions with >200 bases, with a CG content of at least 50%, and with an observed, expected CpG frequency of at least 60% (Obs/Exp CpG = [Number of CpG/(Number of C x Number of G)] x N; where N is the total number of nucleotides in the sequence analyzed). About 70% of human genome promoters are associated with CpG islands, of which approximately 94% remain unmethylated in normal cells [11]. DNA methylation could also occur in regions of lower CpG density that lie in close proximity to CpG islands (~2 kb) called CpG island shores, and their methylation is also associated with gene silencing [12].

**Fig. (1):**
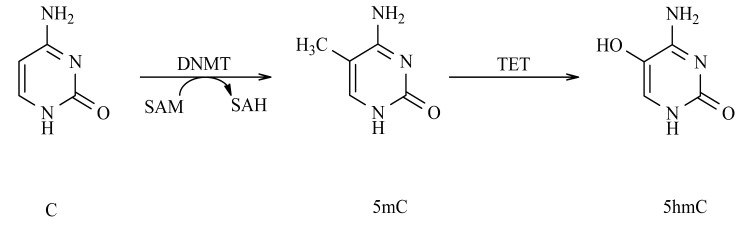
DNA methylation and DNA hydroxymethylation. DNMTs catalyses the transfer of a methyl group from SAM to the fifth carbon of C creating 5mC. The methyl group of 5mC can be modified by the addition of a hydroxyl group mediated by TETs to generate 5-hydroxymethyl-cytosine (5hmC). DNMTs: DNA methyltransferases; SAM: S-adenosyl methionine; SAH: S-adenosyl homocysteine; C: cytosine; 5mC: 5-methylcytosine; 5hmC: 5-hydroxymethyl-cytosine; TETs: ten-eleven translocation cytosine dioxygenases

DNA methylation occurs through the covalent addition of a methyl group to C5 position of Cytosine (C) to form methylated Cytosine (5mC). This is mediated by DNA Methyltransferases (DNMT) that catalyze the transfer of the methyl group from S-Adenosyl-Methionine (SAM) to the C base (Fig. **1**). There are two types of DNMT: *de novo* and maintenance. DNMT3A and DNMT3B are the *de novo* DNMT and are those responsible for establishing the pattern of methylation during embryonic development. DNMT1 is the maintenance DNMT and is responsible for maintaining DNA methylation patterns during cell division [13]. DNA methylation can inhibit gene activity directly because the methylation of promoter CpG islands interferes with the binding of Transcription Factors (TF) to their target sites [14]. DNA methylation can also indirectly promote gene silencing by recruiting the Methyl-CpG-Binding Domain (MBD) proteins that form part of Methyl-CpG-binding Protein (MeCP) complexes [15]. Of particular importance are MeCP1 and MeCP2, which mediate transcriptional repression by favoring Histone Deacetylase (HDAC) activity, leading to histone deacetylation [16].

### 3.3 The Role of DNA Methylation in Osteoarthritis

Several studies have investigated the role of DNA methylation in *loci* involved in different pathways or molecules related to OA. The most relevant findings are mentioned and (Table **1**) provides a summary of the individual genes analyzed.

#### 3.3.1 *Methylation in* ECM *genes*

Chick embryo chondrocytes express type II collagen; however, if they dedifferentiate, they cease their synthesis and begin to synthesize type I collagen, which is more typical in fibroblasts. Interestingly, in chick embryo chondrocytes, the *Col2a1* gene exhibits reduced methylation in comparison with that of fibroblasts, but there is no change in methylation status when chondrocytes become dedifferentiated [17]. In human articular chondrocytes and in MSC subjected to chondrogenesis, all of the 74 CpG sites of a region around the Transcription Start Site (TSS) of the *COL2A1* gene promoter are unmethylated. However, this does not correlate with *COL2A1* expression because, in human articular chondrocytes, gene expression is low, whereas in MSC subjected to chondrogenesis, *COL2A1* demonstrates high expression [18]. On the other hand, in chondrocytes from patients with OA and controls, all of the 21 CpG sites in the *COL2A1* enhancer are nearly completely demethylated; however, *COL2A1* expression is 9-fold higher in OA chondrocytes in comparison with controls [19]. All of these findings suggest that the regulation of *COL2A1* could be independent of DNA methylation of the gene itself.

Type IX collagen is important for the formation of a stable collagen network and for the maintenance of cartilage organization and integrity. *COL9A1* gene-expression levels in OA chondrocytes are 6,200-fold lower than in normal chondrocytes, and six of the eight CpG sites of the *COL9A1* promoter are significantly hypermethylated [19]. Human articular chondrocytes are negative for type X collagen unless they become hypertrophic. In these cells, two CpG sites of *COL10A1* are consistently methylated and there is no gene expression. In contrast, in chondrocytes produced from MSC, there is reduced methylation at these two CpG sites, with strong *COL10A1* expression [18]. Aggrecan is an essential component of cartilage ECM that is reduced during aging; hence, it is associated with OA development; however, there are no significant changes in methylation levels at the CpG island of the Aggrecan (*ACAN*) gene promoter in normal aged or in OA chondrocytes [20]. Thus, it appears that the expression of *ACAN* is not modulated by changes of methylation in the promoter region.

#### 3.3.2 Matrix-Degrading Enzyme Genes

MMP expression in healthy cartilage is low and, in contrast, it is elevated in OA, resulting in ECM degradation. Methylation analysis of the promoter region of *MMP3*, *MMP9*, and *MMP13* genes in OA cartilage shows a significant loss of methylation in comparison with normal cartilage. However, not all CpG sites of these promoter genes are equally susceptible to methylation loss and, for each gene, there is a specific CpG site from TSS where demethylation is more significant: at -635 bp for *MMP3*; at -36 bp for *MMP9*, and at -110 bp for *MMP13* [21]. In addition, methylation at the -110 bp site for *MMP13* decreases the binding of Hypoxia-inducible Factor 2 Alpha (HIF-2α), a TF that regulates *MMP13* expression [22]. On the other hand, demethylation of another specific region in the *MMP13* promoter, the -104 bp site, correlates with increased *MMP13* expression and avoids the binding of a TF, the cAMP Response Element Binding (CREB) [23]. ADAMTS5 is considered the major aggrecanase in OA; however, *ADAMTS4* also contributes to aggrecan degradation. There is methylation loss at CpG sites in the *ADAMTS4* promoter, but the -753 bp site comprises the most consistently demethylated site, with concomitant, strong *ADAMTS4* expression up to 700-fold in the surface zone of OA cartilage [21, 24]. All of this is of interest because previously it was widely thought that the methylation of each CpG site was required to repress gene expression; however, these findings suggest that methylation of a single site may be sufficient to affect gene expression.

#### 3.3.3 Inflammation-Related Genes

Under normal conditions, there is no expression of IL-1β gene (*IL1B*) in human articular chondrocytes; however, when these are experimentally exposed to IL-1β and TNF-α there is a loss of DNA methylation at a specific CpG site (-299 bp) in the promoter and *IL1B* expression increases 100- to 1,000- fold [25]. Interestingly, in OA chondrocytes, the same *IL1B* CpG site is demethylated [22], which suggests that inflammatory cytokines can change methylation status at specific CpG sites during the OA process. Interleukin 8 (IL-8), also known as Chemokine (C-X-C motif) Ligand 8 (CXCL8), is a pro-inflammatory chemokine that mediates the activation and migration of neutrophils into tissue from peripheral blood, as well as the release of MMP13 [26]. In OA chondrocytes, *IL8* expression is considerably high, and its promoter region is significantly demethylated at three CpG sites: -116 bp; -106 bp, and -31 bp, with -116 bp CpG site the strongest predictor of *IL8* expression [27]. Suppressors of Cytokine Signaling proteins (SOCS) are inhibitors of cytokine signaling, and include SOCS1-SOCS7 and Cytokine-Inducible SH2-domain-1 (CIS-1), with SOCS1, SOCS2, SOCS3, and CIS-1 the best characterized of these [28]. The *SOCS2* promoter possesses 28 CpG sites in OA chondrocytes; 16 of these CpG sites, located between -920 and -641 bp, are hypermethylated, while 13 CpG sites, localized between -419 and -15 bp, are demethylated. However, there is no difference in *SOCS2* promoter methylation status between OA and normal chondrocytes or in human chondrocytes after cytokine stimulation. On the other hand, the gene expression of *SOCS2* is reduced in OA chondrocytes [29], suggesting that this is not regulated due to DNA methylation of the gene itself.

Leptin (LEP) is a cytokine-like peptide secreted by white adipose tissue that can regulate bone growth through collagen synthesis, mineralization, osteoblast proliferation, and the stimulation of endochondral ossification [8]. LEP is directly correlated with the OA grade, and its upregulation increases *MMP9* and *MMP13* expression [30]. In healthy chondrocytes and in the chondrocytes of minimal OA, *LEP* promoter is highly methylated with a consequent very low gene expression; this downregulation of *LEP* exerts an effect on *MMP13* expression by reducing its levels significantly, whereas in advanced OA, *LEP* promoter possesses very low levels of methylation and the gene is highly expressed [31].

#### 3.3.4 Reactive Oxygen Species-Related Genes

Chondrocytes express Nicotinamide adenine dinucleotide phosphate-Oxidase (NOX) and Nitric Oxide Synthase (NOS) family members, which generate the Reactive Oxygen Species (ROS): NO and anion superoxide. The increase of NO in articular chondrocytes suppresses energy metabolism, activates MMP, and represses the synthesis of collagen type II and aggrecan in ECM [32]. To prevent damage due to ROS accumulation, chondrocytes produce the Superoxide Dismutase (SOD) enzymes SOD1, SOD2, and SOD3, which are downregulated in OA chondrocytes. In OA cartilage, there is significantly increased methylation at CpG sites of the *SOD2* promoter, with low expression levels of the gene [33]. NO production in OA is also the consequence of upregulation of inducible NOS (iNOS), which is activated by IL-1β and TNF-α [34]. In the promoter and enhancer regions of *iNOS,* between -1400 bp and +117 bp from the TSS, there are 13 CpG sites (seven in the promoter, and six in exon 1 nearest to the TSS). Nevertheless, in OA and normal chondrocytes, six of the seven CpG sites of the *iNOS* promoter are predominantly methylated, and the remaining CpG sites of the promoter closest to the TSS (-289 bp) and the six sites of the exon 1 are demethylated [35]. These data indicate that epigenetic regulation of *iNOS* in human chondrocytes does not involve the promoter region between -1400 bp and -117 bp.

*iNOS* expression is regulated by NF-κB, a signaling factor activated by tissue damage and inflammation. The *iNOS* enhancer region contains NF-κB binding sites, and the CpG sites of that region in OA chondrocytes are significantly demethylated. It appears that the loss of methylation at specific CpG sites in the *iNOS* promoter could not account for its abnormal expression; however, demethylation of specific NF-κB enhancer elements could explain the increased *iNOS* expression in OA [35]. Interestingly, glucosamine and an NF-κB inhibitor inhibit cytokine-induced demethylation at a specific site in the *IL1B* gene promoter, resulting in decreased gene expression [36].

#### 3.3.5 Transcription Factor Genes

Sex-determining region Y-box 1 (SOX) belong to a family of TF that carries a characteristic High-Mobility-Group (HMG) domain that binds DNA in a sequence-specific manner [37]. SOX are expressed in all chondrogenic cells, with the exception of hypertrophic chondrocytes, and play a significant role in the maintenance of chondrocytic phenotypes [37]. SOX9 is a key factor for chondrogenesis because it is required for controlling the expression of essential ECM genes, such as *COL2A1*, *COL9A1*, *COL11A1*, and *ACAN* [37, 38]. Methylation analysis of the promoter region of *SOX9* and Runt-related transcription factor 2 (*RUNX2*) in MSC subjected experimentally to chondrogenesis demonstrates low methylation and increased gene expression, regardless of the differentiation status during chondrogenesis [39], whereas in OA chondrocytes, there is downregulation of SOX9 and promoter methylation is increased, reducing the binding affinity of TF such as CREB and CCAAT-Binding Factor/Nuclear Factor-Y (CBF/NF-Y) [40]. These data indicate that promoter regions of chondrogenesis-related genes *SOX9* and *RUNX2* are maintained at low levels during chondrogenesis to favor their expression; however, a change in the methylation status of *SOX9* promoter could be associated with OA development.

#### 3.3.6 Growth Factors and Its Antagonists

Bone Morphogenetic Protein 7 (BMP7), or Osteogenic protein 1 (OP-1), is a member of the TGF-β superfamily of regulatory molecules. BMP7 is involved in cartilage maintenance and repair by regulating genes of ECM, of anabolic pathways and of bone formation, as well as genes of regulation of cytokines and of various catabolic pathways responsible for ECM degradation and apoptosis [41]. In aged chondrocytes, a positive correlation between age and methylation of the *BMP7* promoter is observed, with concomitant decreased expression of *BMP7*, as well as of genes regulated by *BMP7*, such as *IGF-1*, IGF-1 receptor (*IGF-1R*), and *ACAN* [42]. This age-related increased methylation in the *BMP7* promoter may contribute to the cartilage loss observed in aging and during the progression of OA. Sclerostin (SOST) is a BMP antagonist that modulates mitogenic activity through sequestering BMP [43]. In OA, SOST promotes subchondral bone sclerosis and inhibits cartilage degradation [44]. In OA chondrocytes, the CpG region of the *SOST* promoter is hypomethylated and gene expression is upregulated compared with normal chondrocytes. Interestingly, demethylation of the gene promoter favors Smad 1/5/8 binding, increasing the expression of *SOST* [45].

Growth Differentiation Factor 5 (GDF5) is also a member of the TGF-β superfamily and is implicated in chondrogenesis and in chondrocyte proliferation [46]. A Single Nucleotide Polymorphism (SNP) located in the 5´Untranslated Region (5´UTR) of the *GDF5* gene, the rs143383 (C/T), is strongly associated with OA and exerts a functional effect [47]. In OA chondrocytes, the OA-risk T allele exhibits lower expression of the gene than that of the C allele, a phenomenon known as Differential Allelic Expression (DAE) [48]. The effect of rs143383 is dependent on a second SNP also localized within the 5´UTR of *GDF5*, the rs143384 (C/T), and decreased expression of the T allele of rs143383 is only observed in individuals who are compound heterozygous for both SNP. In cell lines and joint tissues, there is demethylation of *GDF5* correlating with its increased expression, and interestingly, CpG sites formed by the C alleles of both SNP are variably methylated, and demethylation of the heterozygous cell line increases the DAE imbalance between C and T alleles [49]. This indicates that the DAE variability, thus the OA susceptibility conferred by rs143383, is regulated by DNA methylation.

#### 3.3.7 Other Genes

Similar findings to those for *SOCS2* are found in the Cyclin-dependent kinase inhibitor 1 (*p21^WAF1/CIP1^*) gene; this is not an inflammation-related gene, but an inhibitor of cell proliferation that is highly expressed in non-proliferating chondrocytes [50]. In OA chondrocytes, *p21^WAF1/CIP1^* is downregulated regardless of its promoter methylation status [51]; thus, it appears that at least in chondrocytes, its downregulation is not due to hypermethylation of the promoter.

The Deionidase Iodothyronine type 2 (*DIO2*) gene encodes a selenoprotein that catalyzes the conversion of an inactive thyroid hormone (T4) to its active form (T3). T3 drives the terminal maturation of growth plate chondrocytes, leading to cell hypertrophy, ECM destruction and mineralization, and the formation of bone [52]. Two SNPs of *DIO2* have been associated with OA, with a protective association conferred by the T allele of rs12885300 (T/C), and a clear, predisposing association with the C allele of rs225014 (C/T) and with the haplotype C-C formed by these two SNP [53]. *DIO2* also exhibits DAE, with the OA-risk C allele more abundantly present in articular joint tissues than the T allele [54]. In the methylation analysis of *DIO2*, a CpG site 2031 bp upstream of the TSS is methylated in OA cartilage, with a surprisingly positive association between methylation and *DIO2* expression. This effect appears to be driven by the risk C allele, because the homozygosity and heterozygosity for this allele show hypermethylation at the CpG 2031 bp site with increased expression of *DIO2*, while in the homozygous T allele, there are no differences in methylation or gene expression [55]. This positive correlation between methylation and *DIO2* expression in articular cartilage among carriers of the rs225014 OA-risk C allele does not act in accordance with the typical inverse relationship between CpG methylation and gene expression.

#### 3.3.8 Other Cytosine Modifications

Most studies have explored the role of C methylation; however, recently a role for 5-hydroxymethylcytosine (5hmC) as an epigenetic mark has been described. The Ten-eleven translocation cytosine dioxygenases (TET) comprise TET1, TET2, and TET3. These proteins catalyze 5mC oxidation and generate 5mC derivatives, including 5hmC, which is stably present in the majority of tissues and might function as an epigenetic mark (Fig. **1**) [56]. A recent study demonstrated a global increase in 5hmC levels up to 5-6-fold in OA chondrocytes, and *locus*-specific analysis showed a significant increase of 5hmC content in *MMP1* and *MMP3* promoters. With regard to TET proteins, there are no differences in TET2 and TET3 expression, but surprisingly, significant downregulation of TET1 is observed in OA chondrocytes, as well as in normal chondrocytes exposed to IL-1β and TNF-α [57]. It is surprising that significant downregulation of TET1 is concomitant to an increase in 5hmC in OA chondrocytes, considering that a loss of TET1 in some cancers leads to an opposite effect: a global loss of 5hmC [58]. These observations suggest that the increase in 5hmC and downregulation of TET1 demonstrate clear perturbation of 5hmC homeostasis in OA chondrocytes.

#### 3.3.9 Genome-wide Methylation Analyses

Lately, genome-wide methylation and genome-wide 5-hydroxymethylation profiles in OA have been developed with very interesting results (Table **2**) [59-66]. The main findings of these studies can be summarized as follows: 1) OA can be distinguished undoubtedly from controls according to their DNA methylation profile; 2) OA of the hip and the knee possesses different DNA methylation profiles; 3) Differentially Methylated Regions (DMR) are represented more consistently in genes participating in TGF-β signaling, developmental pathways (specifically the homeobox family of TF), inflammation, and ECM degradation pathways; 4) Differentially Methylated *Loci* (DML) more consistently include *RUNX1*, genes associated with the degradation of ECM (*ADAMTS* and *MMP*), and genes that are members of the TGF-β signaling pathway (*ACRV1B*, *SMAD2*, *SMAD3*, *TGFBR2*, *TGFB1*, and *BMP6*); 5) there are DMR between OA and patients with Osteoporosis (OP), with an inverse methylation relationship of methylation in both groups; 6) OA can be differentiated from controls according to their 5-hydroxymethylome, observing a global increase of 5hmC, at least for OA of the knee, and 7) Differentially hydroxymethylated Regions (DhMR) are related to Wnt-signaling and bone-remodeling pathways, as well as with genes previously related to OA (*MMP3*, *GDF5*, and *COL11A1*).

## CONCLUSION

Analysis of DNA methylation in individual *loci* in OA chondrocytes has shown the expected correlation between methylation levels and gene expression; however, there are exceptions to this rule, such as the *COL2A1*, *SOCS2*, and *p21^WAF1/CIP1^* genes, in which, despite low methylation levels, gene expression is also decreased. This suggests that, at least for these genes, demethylation is not sufficient to inactivate gene expression, and that several factors could be acting to favor this, for instance, the following: 1) the methylation state of other CpG sites in the islands or even in the island shores of these *loci*; 2) the role of other epigenetic mechanisms in transcriptional repression, such as histone modifications, chromatin remodeling, and miRNA, in that they are not mutually exclusive [67], and 3) the method employed to assess DNA methylation because, in several studies, the method was different and this could confer variations on the results (Table **1**).

According to the findings of genome-wide methylation analyses, OA is differentially methylated in comparison with controls; however; there is evidence that OA of the hip and of the knee possess different epigenetic signatures. The comparison between OA of the knee and of the hip has revealed several differentially methylated loci, and the gene ontology analysis of those genes expose the preponderance of developmental pathways (*i.e*., skeletal system morphogenesis) and *HOX* genes [61, 64, 65]. The latter comprise a highly conserved cluster of genes that encode transcription factors of embryonic development that regulate limb morphogenesis and skeletal formation [68]. The location-specific expression of *HOX* genes might contribute to defining joint-specific biology and suggests that there may be unique pathways that distinguish OA of the knee from OA of the hip. In this regard, it has been described that OA of the knee and of the hip has also different genetic associations [69]. These findings indicate that OA-associated signals are often joint-specific, and it can be proposed that at least these OA types could be considered as two different entities.

Since OA possesses distinctive methylation profiles, DNA methylation profiling could be a useful diagnostic tool, allowing for the identification not only of OA, but also to differentiate knee samples from hip samples, emphasizing the importance of separating the study of OA by anatomical site. On the other hand, several genes involved in OA- specific pathways are differentially methylated may offer potential therapeutic targets. For example, it is well-recognized that IL-1β is implicated in the pathogenesis and progression of OA with an associated CpG demethylation in its gene promoter [35, 38]. Interestingly, in human chondrocyte cultures, it was demonstrated that glucosamine can prevent cytokine-induced demethylation at the -256 CpG site of *IL1B* promoter with a consequent decreased expression of IL-1β [70], suggesting that modification of DNA methylation is a potential therapeutic strategy for intervening in the OA process. Although further evidence is required to confirm these results, this sheds light on advances for future therapeutic approaches to the disease.

Finally, the recent development of genome-wide hydroxymethylation analysis has made evident the role of another epigenetic mark, the 5hmC [66], the so-called sixth base, the role of which should be further analyzed in OA.

## Figures and Tables

**Table 1:** Methylation analysis of individual loci in osteoarthritis.

**Gene**	**Cell**	**Samples**	**Methylation levels**	**Gene expression**	**Strategies for DNA methylation analysis**	**Ref.**
**ECM**						
*COL2A1*	Human chondrocytes	AC of the knee with no abnormalities	L	↓	Restriction enzyme-based method and DNA bisulfite sequencing	[18]
	MSC on chondrogenesis		L	↑		
	OA chondrocytes	THR vs. #NOF	L	↑	Bisulfite pyrosequencing	[19]
*COL9A1*	OA chondrocytes	THR vs. #NOF	H	↓	Bisulfite pyrosequencing	[19]
*COL10A1*	Human chondrocytes	No cartilage abnormalities	H	↓	Restriction enzyme-based method and DNA bisulfite sequencing	[18]
	MSC on chondrogenesis		L	↑		
*ACAN*	OA chondrocytes	Not specified	L	-		[20]
**Proteases**						
*MMP3*	OA chondrocytes	THR vs. #NOF	L	-	Restriction enzyme-based method	[21]
*MMP9*	OA chondrocytes	THR vs. #NOF	L	-	Restriction enzyme-based method	[21]
*MMP13*	OA chondrocytes	THR vs. #NOF	L	-	Restriction enzyme-based method	[21]
	OA chondrocytes	THR vs. #NOF	L	↑	Bisulfite pyrosequencing	[22]
	OA chondrocytes	THR vs. #NOF	L	↑	Bisulfite sequencing and pyrosequencing	[23]
*ADAMTS4*	OA chondrocytes	THR vs. #NOF	L	-	Restriction enzyme-based method	[21]
		THR vs. #NOF	L	↑	Restriction enzyme-based method	[24]
**Inflammation**						
*IL1B*	Human chondrocytes^a^	THR vs. #NOF	L	↑	Restriction enzyme-based method	[25]
	OA chondrocytes	THR vs. #NOF	L	↑	Bisulfite pyrosequencing	[22]
*IL8*	OA chondrocytes	THR vs. #NOF	L	↑	Bisulfite pyrosequencing	[27]
*SOCS2*	OA chondrocytes	THR vs. #NOF	L	↓	Bisulfite pyrosequencing	[29]
*LEP*	Min OA and normal chondrocytes	THR and TKR vs. Normal chondrocytes	H	↓	DNA bisulfite sequencing	[31]
	Max OA chondrocytes		L	↑		
**ROS-related**						
*SOD2*	OA chondrocytes	THR vs. #NOF	H	↓	DNA bisulfite sequencing	[33]
*iNOS*	OA chondrocytes	THR vs. #NOF	L	↑	Bisulfite sequencing or pyrosequencing.	[34]
**Chondrogenesis**						
*SOX9*	MSC on chondrogenesis	Synovium–derived MSC	L	↑	DNA bisulfite sequencing	[39]
	OA chondrocytes	THR vs. #NOF	H	↓	Methylation-specific PCR and bisulfite sequencing	[40]
*SOX4*		Synovium-derived MSC	L	↓	DNA bisulfite sequencing	[39]
*RUNX2*	MSC on chondrogenesis	Synovium-derived MSC	L	↑	DNA bisulfite sequencing	[39]
**Growth factors**						
*BMP7*	Human aged chondrocytes	Knee tissue donors	H	↓	Methylation specific PCR	[42]
*SOST*	OA chondrocytes	TKR vs. Knee fracture	L	↑	Methylation-specific PCR and bisulfite sequencing	[45]
*GDF5*	Human chondrocytes	CH8 cell line	L	↑	Bisulfite pyrosequencing	[49]
**Other**						
*p21^WAF1/CIP1^*	OA chondrocytes	TKR vs. Normal human femoral condyles	L	↓	DNA bisulfite sequencing	[51]
*DIO2*	OA chondrocytes	THR/TKR vs. Preserved chondrocytes	H	↑	MALDI-TOF mass spectrometry	[55]

L: Low; H: High; ECM: Extracellular Matrix; AC: Articular Cartilage; MSC: Mesenchymal Stem Cells; THR: Total Hip Replacement; TKR: Total Knee Replacement; #NOF: Fracture of the Neck Of Femur; ^a^Exposed to IL-1β and TNF-α; ROS: Reactive Oxygen Species.

**Table 2:** Genome-wide methylome and genome-wide 5-hydroxymethylome profiles in osteoarthritis.

**Sample (*n*)**	**CpG sites analyzed**	**DML**			**Gene Ontology Analysis ^a^**	**Ref.**
		**All**	**Hyper-methylated**	**Hypo-methylated**		
THR and Osteoporotic hip (26 vs. 27)	> 27,000	241	217	24	Homeobox superfamily of TF (HOXA9, IRX2, MSX2) and genes of cell differentiation	[59]
TKR and Healthy controls ^b^ (25 vs. 20)	> 27,000	91	54	37	Inflammation and Regulation of transcriptional activity-related genes and *RUNX1* and *MSX1* genes	[60]
THR, TKR and #NOF (23/73 vs 21)	>485,000					[61]
THR vs. #NOF		5,322	2,669	2,653	Degradation of ECM, anabolic/catabolic pathway of cartilage homeostasis, TGF-β pathway	
THR Cluster1 vs. Cluster2^c^		15,239	8,524	6,915	Inflammation, Cartilage degradation, and TGF-β pathways	
TKR Cluster1 vs. Cluster2^c^		5,769	3,000	2,769	Immune response	
THR vs. TKR		5,547	2,598	2,949	Genes involved in OA pathogenesis (*ADAM12*, *ADAMTS5*, *GDF5*)	
THR (24)						[62]
OA vs. Intact cartilage	>485,000	550	172	378	TGF-β pathway genes and *RUNX1* and *FURIN*	
TKR (15)	~244,000	1,214	1,070	144	TGF-β and WNT pathways	[63]
Severe OA vs. Mild OA ^d^					Hypermethylation of genes related with TGF-β (*BMP2*, *BMP4*, *MAPK3*, *SMAD*)	
TKR vs. THR		6,272			Developmental pathways (limb development and skeletal system morphogenesis)	[64]
Damaged vs. Undamaged cartilage (14 vs. 17)					Homeobox (HOX) cluster	
TKR, THR, and controls (5, 6, and 7)	>485,000				Skeletal and embryonic organ system development and Homeobox (HOX)	[65]
TKR vs. Controls		72				
THR vs. Controls		26				
TKR/THR vs. Controls		103				
THR vs. TKR		67				
After removing overlaps		239	112	127		
TKR vs. Ligament reconstruction (4 vs. 4)		70,591^e^	44,288^f^	26,303^g^	Increased DhMR in Wnt-signalling and bone-remodeling pathways	[66]
					Loss of DhMR in cell adhesion, skeletal muscle development	
					Gene expression change in MMpP3, MMP13 and inflammation-related genes	

DML: Differential Methylated Loci; THR: Total Hip Replacement; TKR: Total Knee Replacement; NOF#: Neck of Femur fracture.

a: The main results are presented

b: Obtained at autopsy from cadavers with no macroscopic signs of OA

c: Defined according to unsupervised hierarchical analysis

d: Damaged or undamaged cartilage was assessed macroscopically

e: Differentially hydroxyMethylated Regions (DhMR)

f: Hyper-5-hydroxymethyated

g: Hypo-5-hydroxymethyated.
